# Composite and Blends of PFD and MEH-PPV Conjugated Polymer Nanoparticles: Combined Optical Properties and Quantum Yield Increase by FRET

**DOI:** 10.1021/acsomega.6c07561

**Published:** 2026-09-12

**Authors:** Caio Henrique Viana da Silva, Milton Lopes de Lana Junior, Thiago Cazati, Mariana P. Brandao, Andreza Germana da Silva Subtil

**Affiliations:** † Departamento de Física, Centro de Ciências Exatas e Tecnológicas, 28114 Universidade Federal de Viçosa, Av. P.H. Rolfs, s/n Campus Universitário, Viçosa Minas Gerais 36570-000, Brazil; ‡ Departamento de Física, Instituto de Ciências Exatas e Biológicas, 28115 Universidade Federal de Ouro Preto, Campus Universitário Morro Do Cruzeiro, Ouro Preto, Minas Gerais CEP 35400-000, Brazil; § Departamento de Física, Instituto de Ciências Exatas, Av. Antônio Carlos, 6627, Belo Horizonte, Minas Gerais CEP 31270-901, Brazil

## Abstract

Conjugated Polymer Nanoparticles (CPNs) exhibit photophysical properties that depend strongly on morphology, chain conformations, solvent conditions, synthesis processes, the polymer chain’s length and distribution, and interchain/intrachain interactions. The production of CPNs using combinations of two different conjugated polymers can be used to create nanocomposites, by mixing previously formed CPNs from distinct polymers, and to create blend CPNs, by mixing distinct polymers prior to the formation of the CPNs. These two methods can produce diverse nanoparticle solutions with distinct properties due to the interaction between the chosen conjugated polymers. In this work, we fabricated water-dispersible luminescent CPNs from the combination of two distinct conjugated polymers: Poly­[2-methoxy-5-(2-ethylhexyloxy)-1,4-phenylenevinylene], MEH-PPV, and poly­(9,9-di-n-dodecylfluorene-2,7-diyl), PFD. The CPNs prepared separately and subsequently mixed exhibit the combined optical properties of the individual CPNs. More importantly, our results show that in CPNs fabricated with both polymers mixed prior to the formation, PFD can transfer energy by Förster resonance energy transfer (FRET) to MEH-PPV. The FRET efficiency is highly dependent on the concentration and ratio of the polymers, reaching values of efficiency above 90% for distances of the order of 4 nm. This energy transfer in blend nanoparticle samples (CPN/PFD:MEH-PPV) resulted in an overall enhancement of the photoluminescence quantum yield of the hybrid nanoparticles, reaching values up to two times higher than those measured for pure CPN/MEH-PPV nanoparticles. These findings show that the donor–acceptor ratio controls energy-transfer efficiency by regulating the fraction of interacting donor–acceptor pairs in hybrid conjugated polymer nanoparticles, enabling applications in luminescent nanostructures, sensing, and photonic devices.

## Graphical abstract

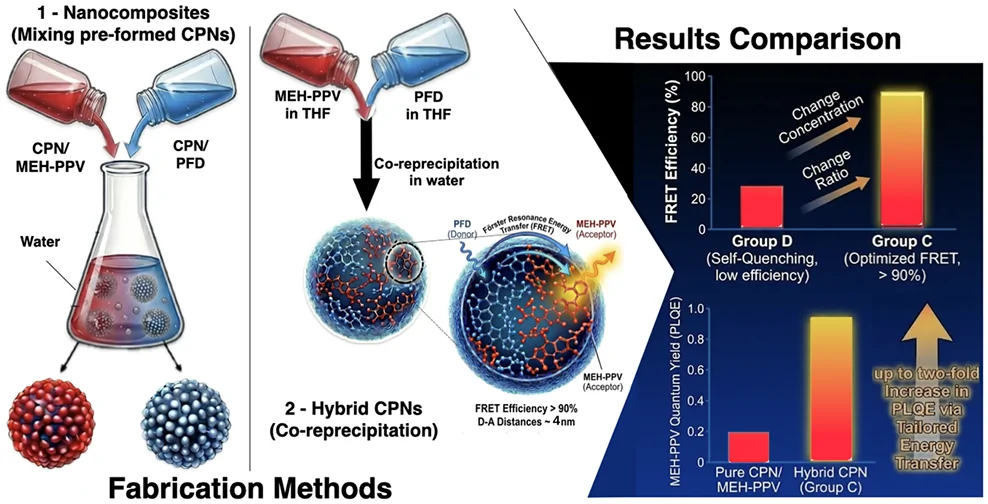

## 1 Introduction

New advancements in nanoscience and nanotechnology have great potential for applications in various fields of knowledge. Materials at the nanoscale can present unique physical, chemical, and biological characteristics, encompassing distinct optical properties that arise naturally with the small size.^1,2^ Nanoscale materials based on conjugated polymers exhibit photophysical properties that depend on morphology and conformations, solvent and synthesis processes, the chain’s length and distribution, and interchain/intrachain interactions.^1^ Moreover, the formation of aggregates and distinct structural phases in the material can change its photophysical properties.^3–5^

Conjugated polymer nanoparticles (CPNs) can be customized for specific purposes by the choice of the conjugated polymers and conjugations with different elements.^6^ In general, semiconducting polymers, which absorb light in a wavelength related to the π–π* energy gap and can present intense fluorescence emission, exhibit photophysical responses strongly dependent on polymer chain conformation. Thus, their photophysical properties are highly influenced by changes along the polymer backbone from an extended to a collapsed state, which can lead to absorption and emission spectral shifts. The close contact between the polymer chains in the collapsed state in CPNs can enhance intra-/interchain energy transfer processes, resulting in modified photophysical properties.^7,8^ The concentration of the conjugated polymer in the solvent before the fabrication of the CPNs can affect the formation of the collapsed state and aggregates of the polymer chains and can also alter the physical properties of the nanoparticles.^9^

Conjugated polymers, especially those involving fluorene-based systems (known as polyfluorenes), are excellent electron and energy donors; this means that the fluorescence of CPNs can be quenched with suitable acceptor materials, which gives them great versatility as sensing elements.^10^ Moreover, these polyfluorenes, such as PFD (poly­(9,9-di-n-dodecylfluorene-2,7-diyl)) and PFO (poly­(9,9-di-n-octylfluorenyl-2,7-diyl)), can exhibit two conformations (α and β phases), in which β conformations (or β phase) have higher intrachain torsion angles resulting in a planar chain structure with a longer conjugated length and well-ordered chains. The formation of β phases in these polymers depends on the degree of aggregation, thus, the polymer’s concentration in the fabrication process of the CPNs can favor β conformations for fluorene-based polymers.^11^ Another widely studied conjugated polymer is poly­[2-methoxy-5-(2-ethylhexyloxy)-1,4-phenylenevinylene] (MEH-PPV), which is well-known for its remarkable optical and electronic properties. MEH-PPV has been extensively employed in organic electronic devices, including organic light-emitting diodes (OLEDs) and organic photovoltaics (OPVs).^12–14^ It exhibits a band gap of approximately 2.3 eV and displays strong photoluminescence in the orange-red region, with emission maxima typically between 550 and 600 nm.^15^ Furthermore, its hydrophobic nature and good solubility in common organic solvents facilitate solution processing, making it particularly attractive for applications in flexible electronics, sensors, and photonic devices.^16–19^

The production of CPNs using combinations of two different conjugated polymers can be used to create nanocomposites, by mixing previously formed CPNs from distinct polymers, and to create blend CPNs, by mixing distinct polymers prior to the formation of the CPNs. These two methods can produce diverse nanoparticles’ solutions with distinct properties due to the interaction between the chosen conjugated polymers. In a previous study,^9^ PFD and MEH-PPV conjugated polymer nanoparticles (CPNs) were synthesized via reprecipitation in THF, revealing that higher polymer concentrations enhance intra-/interchain energy transfer, induce β-phase emission, and increase photoluminescence quantum efficiency (PLQE) in PFD, while MEH-PPV shows saturation.

In this work, we fabricated water-dispersible luminescent conjugated polymer nanoparticles, CPNs, from the combination of two distinct conjugated polymers: MEH-PPV, and PFD. Four groups of CPN solutions were prepared using different fabrication strategies, including blends of polymers within the nanoparticles and mixtures of separately synthesized CPNs. Groups A and B consisted of nanocomposites formed by mixing CPN/PFD and CPN/MEH-PPV at different ratios, whereas Groups C and D comprised hybrid blend nanoparticles (CPN/PFD:MEH-PPV) containing both polymers within the same CPNs at different ratios (Figure 1). Although PFO/MEH-PPV systems have been previously investigated, the corresponding PFD/MEH-PPV system remains largely unexplored, particularly regarding the influence of the nanoparticle fabrication route on FRET efficiency and photophysical properties. Despite sharing the same polyfluorene backbone, PFD differs from PFO by the presence of longer dodecyl side chains instead of octyl substituents. Previous studies have demonstrated that variations in side-chain length significantly affect the self-assembly, intermolecular packing, and aggregate morphology of polyfluorenes, leading to changes in their optical and photophysical properties even without substantial modifications to the polymer backbone.^20^ Consequently, differences in chain packing and intermolecular interactions are expected to influence donor–acceptor distances and, therefore, FRET efficiency. Therefore, the behavior of PFD-based nanoparticles cannot be directly extrapolated from that of PFO-based systems, justifying the investigation of this specific polymer combination. Furthermore, a direct comparison between postmixed nanoparticles and co-reprecipitated blend nanoparticles provides insight into how different assembly routes influence the spatial organization of the two conjugated polymers within the nanoparticles. While postmixing primarily promotes interactions between preformed nanoparticles, co-reprecipitation enables both polymers to assemble simultaneously during nanoparticle formation, potentially leading to different morphologies and interpolymer interactions, which may ultimately affect energy transfer, photoluminescence quantum yield, and emission properties. This comparison provides fundamental insight into how nanoparticle assembly governs the photophysical behavior of hybrid conjugated polymer nanoparticles.

**Figure 1:**
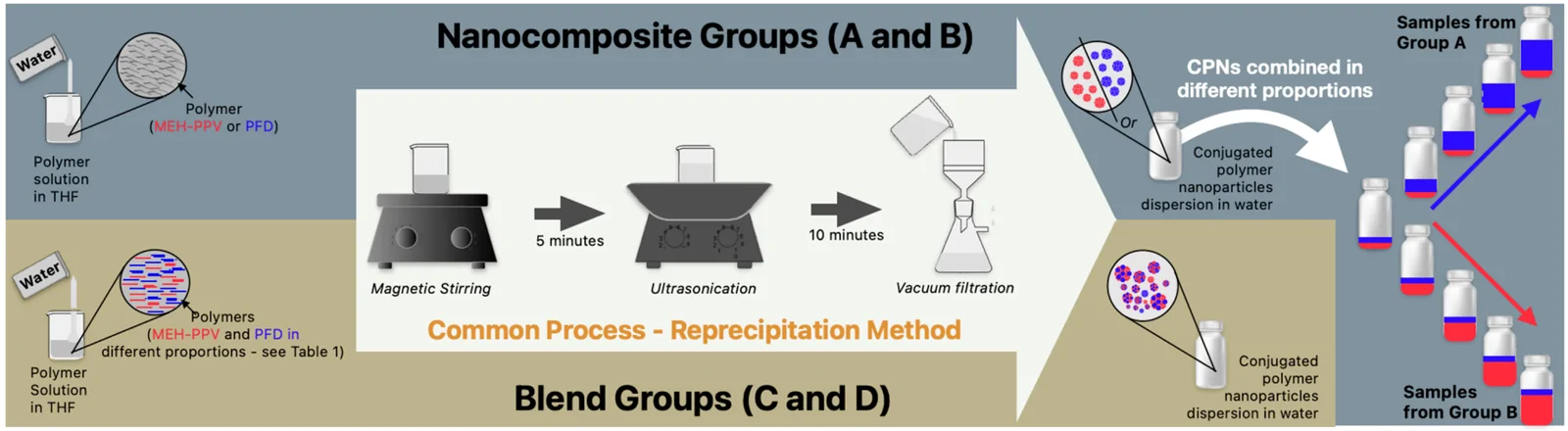
Fabrication process for the nanocomposite samples (Groups A and B) and blend samples (Groups C and D).

The optical and morphological properties of these systems were investigated to compare the interactions between independently formed CPNs in nanocomposites and the interchain interactions within hybrid blend nanoparticles. The nanocomposites exhibited the combined optical properties of the individual CPNs, while the blend CPNs showed Förster resonance energy transfer (FRET) from PFD to MEH-PPV, with efficiencies dependent on the composition ratio, as well as enhanced photoluminescence quantum yield in one group of hybrid samples. The photophysical properties of the isolated CPN/PFD and CPN/MEH-PPV systems reported in our previous work^9^ are used here as reference systems to establish the baseline behavior of the individual donor and acceptor nanoparticles. The focus of the present study is the interaction between these polymers in nanocomposites and hybrid nanoparticles prepared by different fabrication strategies.

## 2 Experimental Section

### 2.1 Materials

The semiconductor polymers PFD (poly­(9,9-di-n-dodecylfluorene-2,7-diyl)) with an average molecular weight of 50,000–150 000 g/mol and MEH-PPV (poly­[2-methoxy-5-(2-ethylhexyloxy)-1,4-phenylenevinylene]) with an average molecular weight of 70,000–100 000 g/mol, as well as tetrahydrofuran (THF), were purchased from Sigma-Aldrich and used without any additional purification. All solutions were prepared by using ultrapure water (Milli-Q).

### 2.2 CPN Synthesis

All CPNs were prepared via the reprecipitation method as described in ref 9. Briefly, a portion of the polymer solution was rapidly injected into a larger volume of water, which served as the nonsolvent. The resulting suspension was magnetically stirred for 5 min and subsequently sonicated in an ultrasonic bath for 10 min to promote nanoparticle formation and improve dispersion. Finally, the suspension was vacuum filtered through a paper filter with a pore size of 2.5 μm to remove large aggregates, yielding a stable aqueous dispersion of CPNs. Four groups of CPN solutions were fabricated: two nanocomposite groups (A and B) and two blend groups (C and D). The nanocomposite groups were obtained by mixing previously synthesized CPN/MEH-PPV and CPN/PFD aqueous dispersions. Both types of nanoparticles were independently prepared via reprecipitation prior to mixing. After nanoparticle formation, the aqueous dispersions were combined according to the PFD:MEH-PPV proportions listed in Table 1, resulting in Groups A and B. In Group A (NCA samples), the concentration of CPN/MEH-PPV was kept constant while the concentration of CPN/PFD was varied, yielding PFD:MEH-PPV ratios from 1:1 to 5:1 (NCA:1–1 to NCA:5–1). In Group B (NCB samples), the concentration of CPN/PFD was maintained constant while that of CPN/MEH-PPV was varied, producing ratios from 1:1 to 1:5 (NCB:1–1 to NCB:1–5).

**Table 1:** All Samples, Divided by Group and Sample Type with the Proportion of PFD–MEH-PPV and the Sample Designation for Each Sample

Sample Type	Group	PFD–MEH-PPV proportion	Sample Designation
**Nanocomposite**	A	1–1	NCA:1–1
		2–1	NCA:2–1
		3–1	NCA:3–1
		4–1	NCA:4–1
		5–1	NCA:5–1
	B	1–1	NCB:1–1
		1–2	NCB:1–2
		1–3	NCB:1–3
		1–4	NCB:1–4
		1–5	NCB:1–5
**Blend Nanoparticles**	C	1–1	BNC:1–1
		2–1	BNC:2–1
		3–1	BNC:3–1
		4–1	BNC:4–1
		5–1	BNC:5–1
	D	1–1	BND:1–1
		1–2	BND:1–2
		1–3	BND:1–3
		1–4	BND:1–4
		1–5	BND:1–5

The blend nanoparticles (Groups C and D) were prepared using a different approach. In this case, MEH-PPV and PFD were first co-dissolved in THF (1 g/L each) according to the proportions given in Table 1. The resulting organic solution was then subjected to the same reprecipitation procedure, maintaining a fixed organic solvent-to-water ratio of 0.1 (v/v), which led to the formation of blend CPNs. For Group C (BNC samples), the MEH-PPV in solution was kept constant while the PFD in solution was varied, generating PFD:MEH-PPV ratios from 1:1 to 5:1 (BNC:1–1 to BNC:5–1). For Group D (BND samples), the PFD in solution was maintained constant while the MEH-PPV in solution was varied, producing ratios from 1:1 to 1:5 (BND:1–1 to BND:1–5). Figure 1 shows a representation of the fabrication process of all samples.

### 2.3 Absorbance and Fluorescence Emission

Absorbance and fluorescence emission spectra were recorded using a HORIBA FluoroMax-PlusC spectrofluorometer for all samples along with their respective polymer solution references, with the solutions placed in quartz cuvettes at room temperature.

### 2.4 Time-Resolved Fluorescence Spectroscopy

Fluorescence intensity decay measurements were carried out using the time-correlated single-photon counting (TCSPC) technique on a FluoTime 200 spectrometer (PicoQuant) equipped with a microchannel plate photomultiplier tube (Hamamatsu R3809-50). Excitation was provided by a 401 nm pulsed diode laser, with repetition rates adjustable between 0.25 and 20 MHz. Fluorescence emission was collected at a right angle to the excitation path, through a polarizer set at the magic angle (54.7°) to eliminate polarization effects. The background signal was negligible. Decay curves were analyzed using FluoFit® software, which applied multiexponential fitting models to the data. Specifically, fluorescence decay profiles were best described by a triexponential decay model, using deconvolution with the instrument response function (IRF), yielding three lifetime components (*τ*_1_, *τ*_2_, and *τ*_3_) and their corresponding normalized pre-exponential factors (*β*_1_, *β*_2_, and *β*_3_, where *β*_1_ + *β*_2_ + *β*_3_ = 1). Fit quality was assessed based on the distribution of weighted residuals and the reduced chi-squared (χ^2^) value. Time-resolved experiments were performed for all samples (Figure S1 presents a sample decay, IRF and fit—see supporting information). The amplitude-weighted (*τ*_*A*_) and intensity-weighted (*τ*_*i*_) fluorescence lifetimes were calculated from the decay parameters using the following equations:^21,22^

$$\tau_{A}=\frac{\underset{i}{\sum }\beta_{i}\tau_{i}}{\underset{i}{\sum }\beta_{i}}\mathrm{and}\tau_{i}=\frac{\underset{i}{\sum }\beta_{i}{\tau_{i}}^{2}}{\underset{i}{\sum }\beta_{i}\tau_{i}}$$

### 2.5 Photoluminescence Quantum Efficiency

The photoluminescence quantum efficiency (PLQE) was determined using the integrating sphere method.^23,24^ For this purpose, we employed an integrating sphere from Newport Spectralar (PTFE), a Coherent Cube Laser operating at 378 nm, and a USB4000 VIS-NIR spectrometer from Ocean Optics. The procedure involved three main measurements: (1) placing the sample at the center of the sphere for direct excitation, capturing both the emission spectrum of the sample (*E*_*i*_) and the laser (*L_i_*); (2) positioning the sample off-center for secondary excitation, recording the oblique emission spectra of the sample (*E_o_*) and the laser (*L_o_*); and (3) measuring the laser spectrum alone with the empty sphere (*L_e_*). Each sample was analyzed in triplicate. The integrated areas under each spectrum were then used to calculate the absorbance (*A*) and the PLQE (*φ_PL_*) using eqs 1 and 2. The PLQE of each sample was determined by the average of triplicate samples with the respective standard deviations.

1 $$A=\frac{L_{o}\left(\lambda \right)-L_{i}\left(\lambda \right)}{L_{o}\left(\lambda \right)}$$

2 $$\varphi_{PL}=\frac{E_{i}\left(\lambda \right)-\left(1-A\right)E_{o}\left(\lambda \right)}{L_{e}\left(\lambda \right)A}$$

### 2.6 Morphological Characterizations

The hydrodynamic mean diameter was determined using dynamic light scattering (DLS) with a Zetasizer Nano S (Malvern Instruments). Measurements were performed in polystyrene cuvettes at a precisely maintained temperature of 25.0 ± 0.1 °C. Scattered light was detected at an angle of 173° with respect to the incident 4 mW He/Ne laser beam (λ = 632.8 nm). The instrument’s software, utilizing the CONTIN algorithm, was used to analyze the intensity autocorrelation functions and derive the particle size distribution. Each reported value represents the average of five sets of multiple measurements taken over a 10 min period. This procedure was repeated three times independently to verify reproducibility and calculate the standard deviation.

Morphological characterization was also carried out using a Field-Emission Gun Scanning Electron Microscope (FEG-SEM, Tescan Mira). For sample preparation, 20 μL of the nanoparticle suspension was deposited onto a silicon substrate and left to dry thoroughly. After complete drying, a thin copper coating was applied to the samples via sputter deposition. The samples prepared were then examined using FEG-SEM.

## 3 Results and Discussion

The normalized absorption and emission spectra of CPN/PFD and CPN/MEH-PPV in water are shown in Figure 2. CPN/PFD exhibits a broad absorption band extending from 300 to 600 nm, with a maximum at 390 nm. Its emission spectrum displays three main vibronic peaks at 434, 460, and 491 nm. Based on results of similar polyfluorene polymers in films and nanoparticles, we can infer that PFD’s absorption is related to the combination of disorderly chain segments with β-phase configurations.^9,25^ The fluorescence vibronic peaks in the blue region have fast lifetimes that can vary from 20 to 300 ps, and the low-energy broad emission (∼530 nm) from interchain transitions attributable to excimers presents longer lifetimes (>450 ps).^11,26,27^ In contrast, CPN/MEH-PPV exhibits a broad absorption band, with a maximum at 475 nm and a shoulder at 335 nm, corresponding to the π → π* transition of the conjugated polymer backbone.^9,25^ Its fluorescence spectrum presents two vibronic peaks at 588 and 635 nm attributed to intrachain and interchain processes with lifetimes shorter than 100 ps and between 100 and 500 ps, respectively. Additionally, MEH-PPV exhibits a low-energy emission around 700 nm attributed to excimers and aggregates, with lifetimes that can exceed 1 ns.^9,28–30^ Importantly, the spectral overlap between the emission of CPN/PFD and the absorption of CPN/MEH-PPV provides favorable conditions for Förster resonance energy transfer (FRET) from CPN/PFD to CPN/MEH-PPV, making these polymers an attractive donor–acceptor pair for investigating energy-transfer processes in hybrid nanoparticle systems (Figure S2 shows FRET spectral overlap—see supporting information).

**Figure 2:**
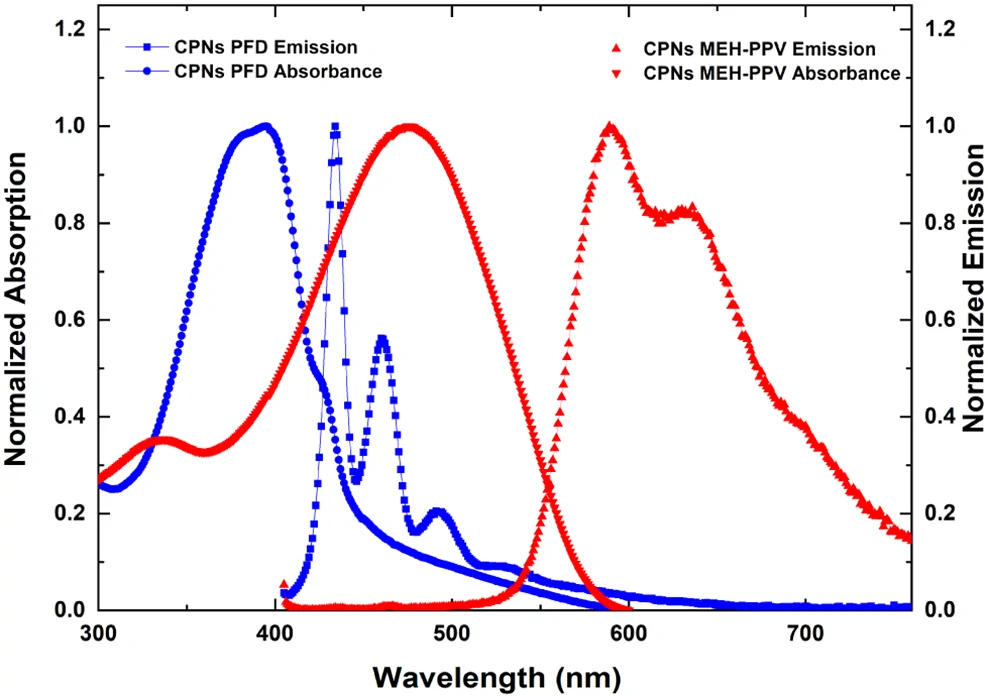
Normalized absorption and normalized emission spectra of CPN/PFD (blue) and CPN/MEH-PPV (red) in water. The samples were excited at 378 nm.

Figure 3 presents the normalized absorption and fluorescence emission spectra for Groups A and B. The absorption spectra were normalized to the maximum absorption peak of the polymer with a fixed concentration. As shown in Figure 3a and b, increasing the proportion of CPN/PFD in Group A leads to a relative increase in the absorption at 380 nm, corresponding to the absorption of PFD, and to an increase in the emission intensity in the spectral region associated with PFD emission. No substantial emission was observed in the spectral region associated with CPN/MEH-PPV emission (550–700 nm), suggesting the absence of energy transfer from PFD to MEH-PPV. These results indicate weak interparticle coupling between the CPN/PFD and CPN/MEH-PPV nanoparticles. Similar to Group A, increasing the proportion of CPN/MEH-PPV (in Group B) leads to a relative increase in absorption at 475 nm, corresponding to the absorption of MEH-PPV (see Figure 3c). However, increasing the proportion of CPN/MEH-PPV results in a decrease in the emission intensity in the spectral region associated with PFD emission, while emission in the red region, associated with MEH-PPV emission, becomes evident but remains essentially unaffected by further increases in the proportion of CPN/MEH-PPV (see Figure 3d). The MEH-PPV emission observed in Group B upon excitation at 378 nm may arise from the absorption of CPN/MEH-PPV in the 300–400 nm region, corresponding to the shoulder of its absorption spectrum. Therefore, the absorption and emission characteristics of Groups A and B indicate the absence of strong interparticle coupling, since the optical response of these nanocomposite systems is dominated by individual contributions rather than efficient donor–acceptor interactions.

**Figure 3:**
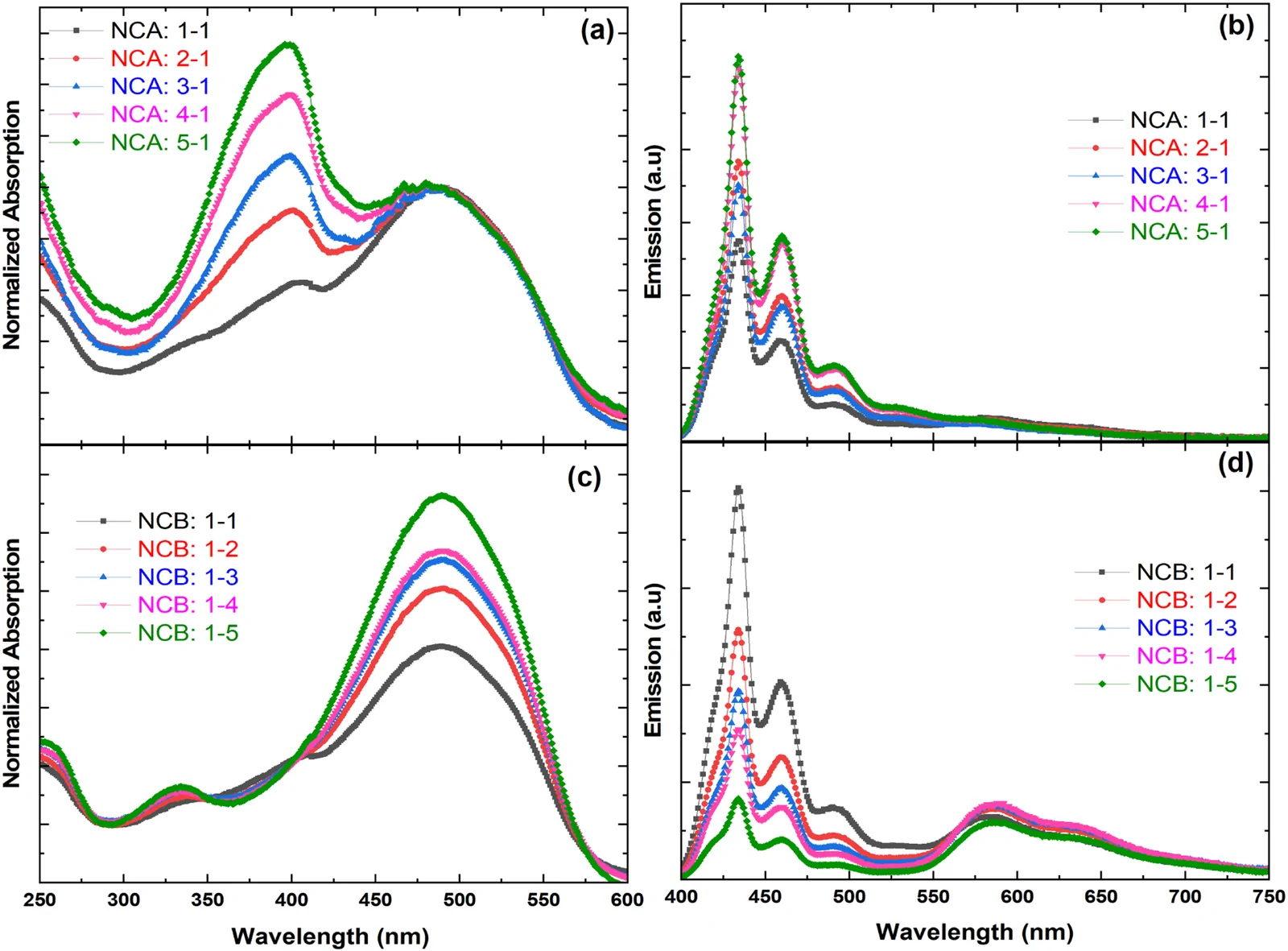
(a) Normalized absorption spectra of Group A; (b) fluorescence emission spectra of Group A; (c) normalized absorption spectra of Group B; and (d) fluorescence emission spectra of Group B. All samples were excited at 378 nm.

To further verify the weak interaction between the CPNs in Groups A and B, photoluminescence quantum efficiency (PLQE) measurements were performed, and the results are presented in Figure 4. Notably, although both groups consist of nanocomposite materials, Group A, which contains a higher proportion of CPN/PFD, exhibits a higher quantum efficiency compared to Group B, which is richer in CPN/MEH-PPV. It is important to highlight that for the 1:1 ratio sample in both groups (NCA:1–1 and NCB:1–1), comprising approximately equal amounts of CPN/MEH-PPV and CPN/PFD solutions, no significant difference in quantum efficiency was observed, as expected. At this composition, the PLQE values for both groups are approximately 0.36%.

**Figure 4:**
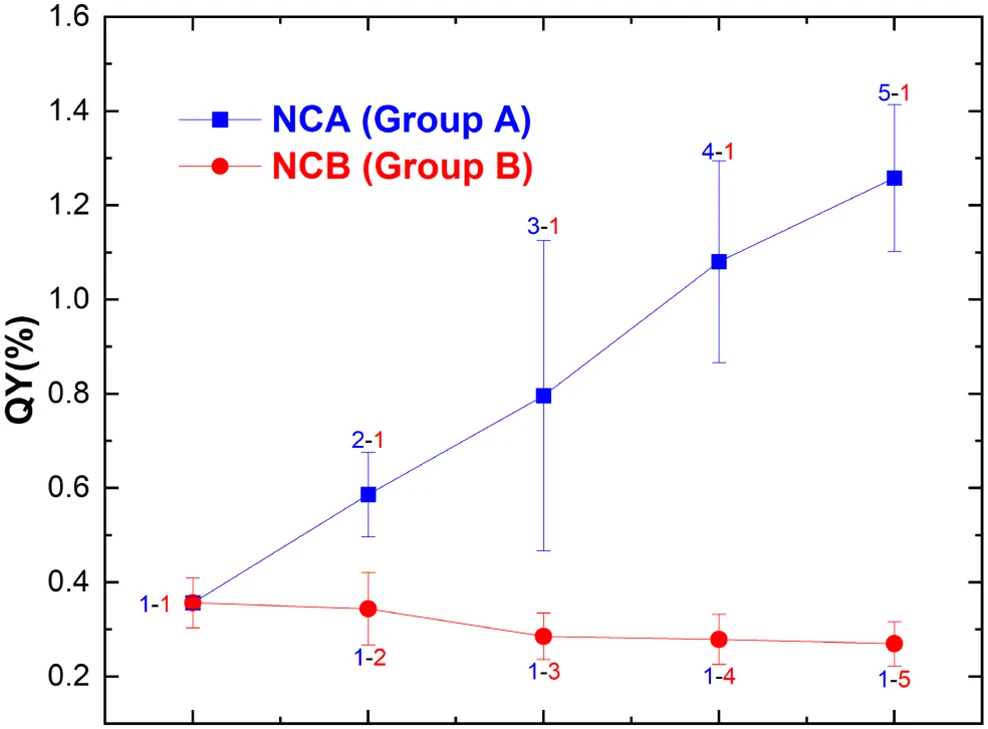
Photoluminescence quantum efficiency of samples from Groups A and B measured using the integrating sphere method. The lines connecting the data points serve only as visual guides. The integrated areas were calculated over the 400–750 nm emission range after excitation at 378 nm.

The error bars shown in Figure 4 correspond to the mean deviation from repeated measurements conducted on different days for all samples, with the experimental setup carefully realigned in a consistent manner for each session. As observed in previous work,^9^ CPN/PFD nanoparticles exhibit photoluminescence quantum efficiencies approximately five times greater than those of isolated CPN/MEH-PPV nanoparticles, due to each polymer’s optical characteristics. Thus, in Group A, the gradual increase in CPN/PFD content during nanocomposite formation likely contributes directly to the overall higher PLQE observed compared to Group B. In contrast, the gradual increase in CPN/MEH-PPV leads to a slight decrease in the photoluminescence quantum efficiency of Group B. Since PLQE corresponds to the ratio of emitted to absorbed photons, this behavior is consistent with the absorption and emission characteristics of Group B, where increasing the proportion of CPN/MEH-PPV enhances the absorption at 475 nm while the MEH-PPV emission remains essentially unchanged with further increases in CPN/MEH-PPV content. These observations further support the interpretation that interparticle coupling in the nanocomposite systems is limited, with the PLQE behavior being predominantly governed by the relative contribution of each nanoparticle population rather than by efficient donor–acceptor energy transfer.

To further investigate the interaction between the CPNs in Groups A and B, time-resolved fluorescence measurements were performed using the TCSPC method for all samples at different concentrations. The fluorescence lifetimes were obtained by multiexponential deconvolution fitting of the decay curves. Table 2 provides the values for the lifetimes *τ*_1_, *τ*_2_, and *τ*_3_, the normalized pre-exponential factors *β*_1_, *β*_2_ and *β*_3_, and the intensity-weighted fluorescence lifetime *τ*_*i*_ and the amplitude-weighted fluorescence lifetime *τ*_*A*_ for Groups A and B. The fluorescence decay curves were fitted using a triexponential model, as it provided the best representation of the experimental data, yielding randomly distributed residuals and satisfactory χ^2^ values. The presence of three lifetime components is consistent with the heterogeneous photophysical behavior typically observed in conjugated polymer nanoparticles, where different emissive environments arising from variations in chain conformation, aggregation state, and intermolecular interactions contribute to the overall fluorescence decay. Depending on the system, these interactions may include aggregate formation, excimer emission, or exciplex formation in nanoparticles composed of different interacting polymers. Therefore, the three lifetime components are associated with distinct photophysical relaxation pathways that contribute to the observed fluorescence decay. The reduced chi-squared (χ^2^) value presents average values of 0.99 ± 0.02 for Group A and of 0.98 ± 0.04 for Group B. Both groups exhibited three distinct lifetimes for emission at 485 nm (PFD emission region). The lifetimes in the ranges of 0.66–0.30 ns and 0.075–0.024 ns are very similar to those previously reported for CPN/PFD in THF.^9^ The intermediate lifetime of approximately 0.145 ns observed for Group A and the longer lifetimes (>1.5 ns) observed for Group B are attributed to excimers and polymer aggregates, which become more significant in the nanoparticle state.^26,27^ Similarly, both groups exhibited three distinct lifetimes for emission at 650 nm (MEH-PPV emission region). The lifetimes of approximately 0.200 ns and 0.050 ns are very similar to those previously reported for CPN/MEH-PPV in THF,^9^ while the lifetimes longer than 0.8 ns observed for Groups A and B are attributed to excimers and polymer aggregates.

**Table 2:** Time-Resolved Fluorescence Parameters of Group A and B Nanocomposites^a^

Samples	*β*_1_ (%)	*τ*_1_ (ns)	*β*_2_ (%)	*τ*_2_ (ns)	*β*_3_ (%)	*τ*_3_ (ns)	*τ*_*A*_ (ns)	*τ*_*i*_ (ns)
Excitation: 401 nm Emission: 485 nm: PFD emission.								
NCA:1–1	12.33	0.145 ± 0.004	1.60	0.66 ± 0.02	86.07	0.0242 ± 0.0007	0.0493	0.2044
NCA:2–1	10.20	0.131 ± 0.003	2.01	0.46 ± 0.01	87.79	0.0211 ± 0.0005	0.0412	0.1569
NCA:3–1	11.67	0.151 ± 0.004	1.72	0.56 ± 0.01	86.61	0.0238 ± 0.0006	0.0479	0.1787
NCA:4–1	13.58	0.158 ± 0.004	1.90	0.60 ± 0.02	84.52	0.0270 ± 0.0008	0.0557	0.1957
NCA:5–1	13.06	0.139 ± 0.003	2.24	0.51 ± 0.01	84.71	0.0241 ± 0.0006	0.0500	0.1765
NCB:1–1	6.02	0.34 ± 0.01	0.41	2.0 ± 0.1	93.57	0.075 ± 0.001	0.1000	0.2978
NCB:1–2	6.15	0.30 ± 0.01	0.68	1.55 ± 0.08	93.17	0.073 ± 0.001	0.0975	0.2767
NCB:1–3	5.35	0.34 ± 0.01	0.53	1.8 ± 0.1	94.12	0.075 ± 0.001	0.0985	0.2940
NCB:1–4	6.62	0.40 ± 0.01	0.63	1.63 ± 0.09	92.75	0.071 ± 0.001	0.0965	0.2827
NCB:1–5	5.37	0.35 ± 0.01	0.42	2.0 ± 0.2	94.21	0.075 ± 0.001	0.0980	0.2930
Excitation: 401 nm Emission: 650 nm: MEH-PPV emission.								
NCA:1–1	1.76	0.82 ± 0.02	22.27	0.181 ± 0.003	75.98	0.0442 ± 0.008	0.0833	0.2331
NCA:2–1	1.71	1.01 ± 0.03	22.51	0.208 ± 0.004	75.77	0.051 ± 0.001	0.1024	0.2844
NCA:3–1	2.20	0.93 ± 0.03	23.52	0.201 ± 0.005	74.28	0.049 ± 0.001	0.1044	0.2912
NCA:4–1	2.30	1.19 ± 0.03	21.59	0.238 ± 0.005	76.11	0.057 ± 0.001	0.1221	0.3883
NCA:5–1	2.36	1.25 ± 0.03	20.60	0.239 ± 0.006	77.05	0.054 ± 0.002	0.1203	0.4233
NCB:1–1	0.41	0.9 ± 0.1	3.90	0.38 ± 0.02	95.69	0.070 ± 0.001	0.0853	0.1570
NCB:1–2	0.31	0.9 ± 0.1	5.61	0.37 ± 0.01	94.09	0.078 ± 0.001	0.0970	0.1640
NCB:1–3	0.32	0.9 ± 0.1	6.16	0.36 ± 0.01	93.52	0.080 ± 0.001	0.1000	0.1650
NCB:1–4	22.79	0.14 ± 0.05	4.37	0.43 ± 0.01	72.85	0.083 ± 0.001	0.0794	0.1638
NCB:1–5	0.21	0.9 ± 0.1	5.15	0.39 ± 0.01	94.64	0.080 ± 0.001	0.0980	0.1600

a *β*_n_ (%): fractional amplitudes; *τ*_n_ (ns): lifetime; *τ_A_* (ns): amplitude average lifetime; *τ_i_* (ns): intensity average lifetime.

The intensity-weighted fluorescence lifetime *τ*_*i*_ and the amplitude-weighted fluorescence lifetime *τ*_*A*_ for Group A emissions at 650 nm (MEH-PPV emission) show a slight increase, suggesting that radiative energy transfer from CPN/PFD to CPN/MEH-PPV may occur. In this process, the fluorescence decay becomes slower due to successive reabsorption and re-emission events involving photons within the spectral overlap region between the donor emission and acceptor absorption spectra. Additionally, no systematic shortening of the donor lifetime associated with CPN/PFD emission was observed, further supporting the absence of nonradiative FRET between nanoparticles prepared separately and mixed after formation, as expected. When Förster resonance energy transfer (FRET) occurs, the fluorescence decay of the donor becomes faster because an additional nonradiative deactivation pathway is introduced through energy transfer to the acceptor. It is important to point out that excitation at 401 nm can be absorbed by both polymers and a small contribution of MEH-PPV emission can be observed in PFD emission analysis and *vice versa*.

Dynamic light scattering (DLS) measurements of the average hydrodynamic diameter for both Groups A and B are presented in Table 3. For Group A, in which the amount of CPN/PFD solutions increases, a slight increase in hydrodynamic diameter is observed in comparison to Group B, where the concentration of CPN/MEH-PPV is increased. Although isolated CPN/MEH-PPV samples exhibited slightly larger hydrodynamic diameters,^9^ the DLS technique relies on multiple average measurements to yield a result.

**Table 3:** Average Hydrodynamic Diameter of the Nanocomposites from Groups A and B

Samples	Diameter (nm)	Samples	Diameter (nm)
NCA:1–1	130 ± 14	NCB:1–1	152 ± 9
NCA:2–1	146 ± 11	NCB:1–2	142 ± 4
NCA:3–1	143 ± 23	NCB:1–3	140 ± 4
NCA:4–1	149 ± 23	NCB:1–4	137 ± 3
NCA:5–1	151 ± 26	NCB:1–5	137 ± 3

Figure 5 presents the absorption and emission spectra of the CPNs obtained from polymer blend solutions (Groups C and D). All absorption spectra were normalized to the maximum absorption peak of the polymer with a fixed concentration. As shown in Figure 5a, increasing the proportion of PFD in the nanoparticles of Group C leads to a relative increase in the absorption at 380 nm, corresponding to the absorption of PFD. Additionally, increasing the proportion of PFD suppresses the emission in the spectral region associated with PFD emission, while the emission associated with MEH-PPV in the 550–700 nm range increases in intensity (Figure 5b). This behavior may be attributed to an effective energy transfer from the PFD species (donor) to the MEH-PPV species (acceptor). It is likely that the collapsed nanoparticle morphology, which promotes closer proximity between different polymer chains, favors interspecies energy transfer and thereby enhances emission at longer wavelengths associated with MEH-PPV. Additionally, the emission spectra, in Figure 5b, reveal a slight red shift in the emission peak of the BNC:1–1 ratio sample compared to the other samples. This behavior may be associated with differences in the molecular organization of the polymer chains within the nanoparticles. Such structural rearrangements may also be reflected in changes in fluorescence anisotropy, indicating a distinct organization of the polymer chains and their transition dipole moments. These rearrangements can modify excitonic coupling and energy relaxation pathways and may increase the effective conjugation length, favoring emission from lower-energy states and resulting in the observed red shift. A similar effect related to anisotropy was observed in pure MEH-PPV thin films.^31,32^ It is important to emphasize that the hybrid polymer solutions in THF prior to the formation of the nanoparticle do not present this abrupt decrease in the PFD emission (Figure S3—see supporting information), suggesting that the collapsed structure obtained by the nanoparticle formation favors Förster resonance energy transfers (FRET) between the polymers’ chains. This behavior contrasts markedly with that observed for nanocomposite systems (Groups A and B), since a small distance between polymer chains is essential for achieving efficient donor–acceptor coupling.

**Figure 5:**
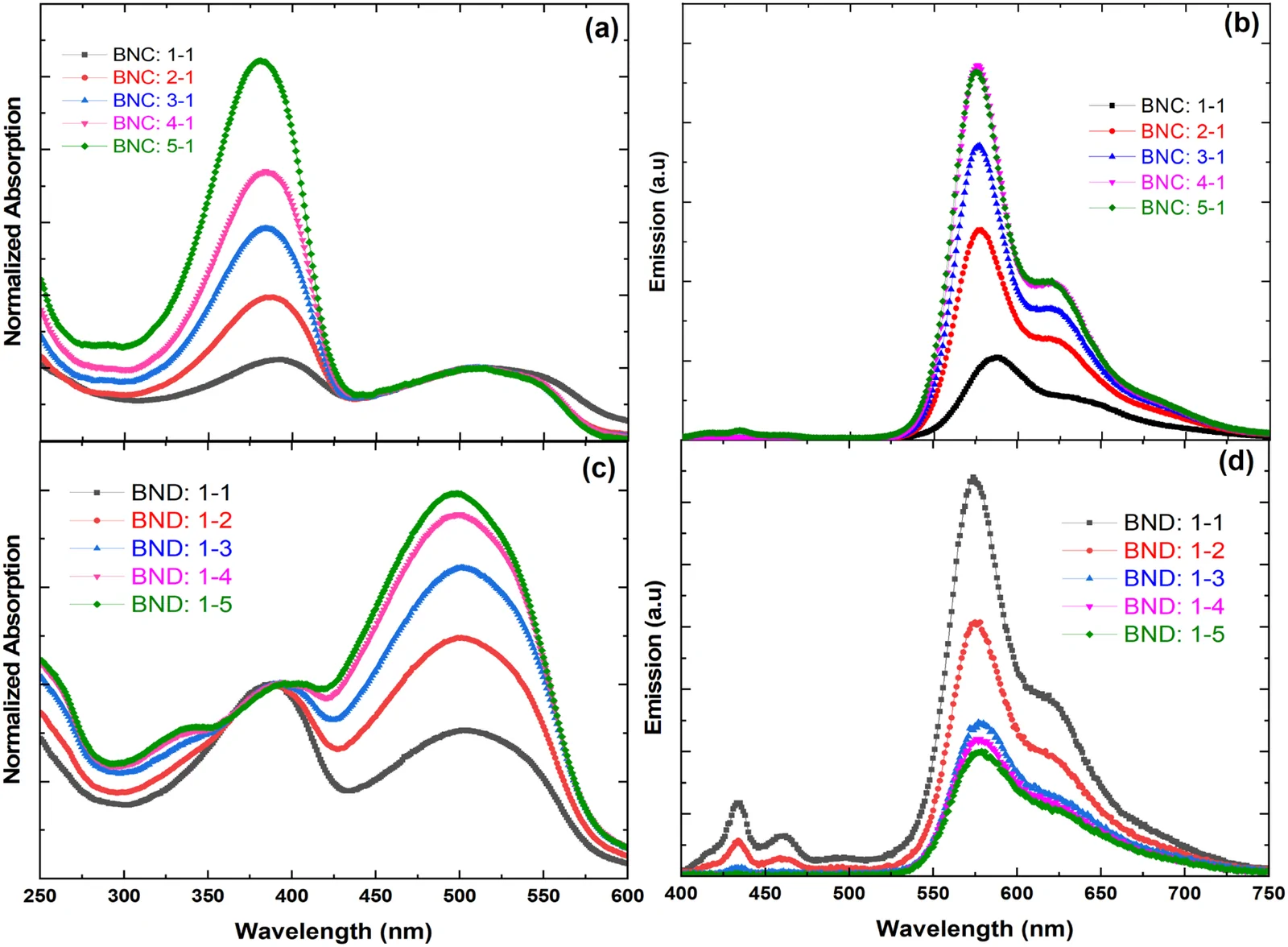
(a) Normalized absorption spectra of Group C; (b) fluorescence emission spectra of Group C; (c) normalized absorption spectra of Group D; and (d) fluorescence emission spectra of Group D. All samples were excited at 378 nm.

Figure 5c and 5d presents the normalized absorption and emission spectra of Group D, in which the concentration of PFD remains constant while the proportion of MEH-PPV is progressively increased. The absorption spectra show a clear increase in the MEH-PPV spectral region with increasing MEH-PPV proportion in the nanoparticles of Group D (see Figure 5c). The emission spectra of Group D, shown in Figure 5d, exhibit dual emission, consisting of a red emission associated with MEH-PPV and a small blue emission associated with PFD. Additionally, increasing the MEH-PPV proportion results in an overall decrease in the emission intensity across the entire spectral range. This behavior may be attributed to the inefficient energy transfer from PFD to MEH-PPV. In addition, part of the decrease in emission intensity may originate from the redistribution of the absorbed excitation resulting from the increased absorption by MEH-PPV at the excitation wavelength (378 nm) together with composition-dependent changes in interchain interactions and excited-state relaxation pathways. As the concentration of the acceptor species (MEH-PPV) increases while the donor concentration (PFD) remains constant, the blue emission associated with PFD decreases; however, unlike Group C, this reduction is not accompanied by a corresponding increase in the red emission from MEH-PPV. Consequently, the emission intensities of both polymers decrease, suggesting that the additional MEH-PPV does not effectively harvest the excitation energy transferred from PFD. Instead, the excess MEH-PPV molecules may promote nonradiative deactivation processes that compete with fluorescence emission, such as self-absorption and energy migration among MEH-PPV chains, leading to a decrease in emission intensity. As also observed in Groups A and B, and demonstrated in ref 9, CPN/PFD samples individually exhibit PLQE values approximately five times higher than those of isolated CPN/MEH-PPV.

The effective energy transfer from PFD (donor) to MEH-PPV (acceptor) in Group C, and the inefficient energy transfer observed in Group D, is reflected in the photoluminescence quantum efficiency (PLQE) behavior of the hybrid nanoparticle systems, as shown in Figure 6. The higher proportion of PFD in Group C’s hybrid nanoparticles, driven by the gradual increase in PFD content, likely contributes only marginally to its higher PLQE compared to Group D (starting from the 1:2 sample), due to the very low emission in the PFD region (see Figure 6). Moreover, increasing the PFD concentration in Group C enhances the nonradiative energy transfer from PFD to MEH-PPV, resulting in stronger MEH-PPV emission and, consequently, higher PLQE values. In contrast, increasing the MEH-PPV concentration in Group D leads to less efficient energy transfer from PFD to MEH-PPV and promotes additional processes, such as self-absorption and energy migration among MEH-PPV chains, which contributes to a reduction in PLQE.

**Figure 6:**
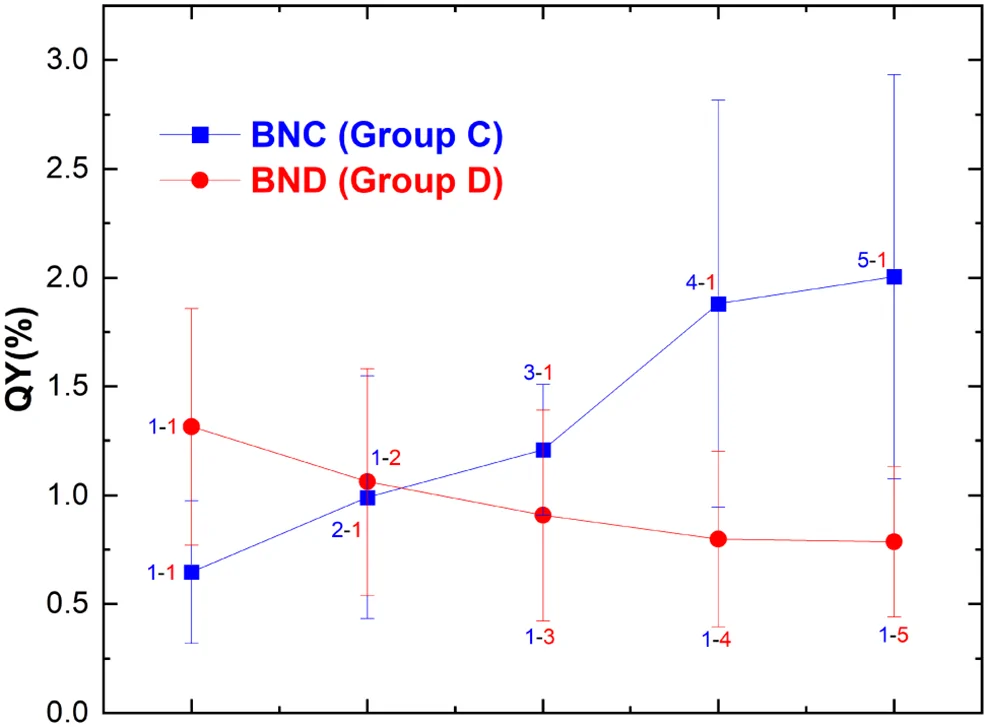
Photoluminescence quantum efficiency of samples from Groups C and D measured using the integrating sphere method. The lines connecting the data points serve only as visual guides. The integrated areas were calculated over the 400–750 nm emission range after excitation at 378 nm.

Unlike the nanocomposite groups, the BNC:1–1 and BND:1–1 samples from these two groups do not exhibit identical PLQE values. This discrepancy is likely related to differences in the preparation methods of the polymeric blends prior to nanoparticle formation. In Group C, the MEH-PPV solution in THF is kept constant with the PFD solution subsequently added on top. Conversely, in Group D, the PFD solution remains fixed, while the MEH-PPV solution is added later. These different deposition sequences may influence how the two polymer species interact during nanoparticle formation, as reflected not only in the quantum efficiency results but also in the time-resolved photoluminescence measurements discussed below.

These results indicate that the donor–acceptor ratio plays a critical role in determining the efficiency of FRET processes and the resulting enhancement of MEH-PPV emission within hybrid nanoparticles. Group C exhibited an increase in total PLQE, which provides independent evidence for efficient FRET within hybrid nanoparticles formed by pre-mixing the polymers before reprecipitation.

The interaction between the polymers in Groups C and D was further investigated by time-resolved fluorescence measurements using the TCSPC method. Fluorescence decay curves were acquired for all samples at different composition ratios, and the corresponding lifetimes were obtained through multiexponential deconvolution fitting. Table 4 provides the values for the lifetimes *τ*_1_, *τ*_2_, and *τ*_3_, the normalized pre-exponential factors *β*_1_, *β*_2_, and *β*_3_, the intensity-weighted fluorescence lifetime *τ_i_,* and the amplitude-weighted fluorescence lifetime *τ*_*A*_ for Groups C and D. The reduced chi-squared (χ^2^) presented average values of 0.97 ± 0.05 for Group C and of 0.99 ± 0.02 for Group D. Similar to Groups A and B, the blend nanoparticles exhibited three distinct lifetimes that reflect the combined fluorescence decay characteristics of both polymers, depending on the emission region analyzed. In addition, faster lifetime components were observed, which are attributed to nonradiative energy transfer processes, while longer lifetime components are associated with radiative energy transfer and possible exciplex emission.^33^

**Table 4:** Time-Resolved Fluorescence Parameters for Group C and D Blend Nanoparticles^a^

Samples	*β*_1_ (%)	*τ*_1_ (ns)	*β*_2_ (%)	*τ*_2_ (ns)	*β*_3_ (%)	*τ*_3_ (ns)	*τ*_*A*_ (ns)	*τ*_*i*_ (ns)
Excitation: 401 nm Emission: 485 nm: PFD emission.								
BNC:1–1	3.35	0.25 ± 0.02	0.24	3.0 ± 0.2	96.42	0.0188 ± 0.0008	0.0336	0.686
BNC:2–1	0.99	0.23 ± 0.02	0.13	2.3 ± 0.2	98.88	0.0101 ± 0.0003	0.0154	0.5004
BNC:3–1	0.81	0.15 ± 0.01	0.09	1.44 ± 0.09	99.10	0.0081 ± 0.0002	0.0105	0.2033
BNC:4–1	0.57	0.20 ± 0.01	0.05	1.3 ± 0.1	99.39	0.0091 ± 0.0002	0.0108	0.1036
BNC:5–1	0.47	0.107 ± 0.008	0.07	0.59 ± 0.04	99.46	0.0062 ± 0.0008	0.0071	0.0473
BND:1–1	3.55	0.135 ± 0.007	0.20	1.19 ± 0.09	96.25	0.01332 ± 0.0004	0.0199	0.1801
BND:1–2	3.28	0.17 ± 0.01	0.20	1.9 ± 0.1	96.52	0.01693 ± 0.0005	0.0259	0.3396
BND:1–3	3.42	0.172 ± 0.008	0.19	2.4 ± 0.2	96.39	0.01690 ± 0.0004	0.0268	0.4762
BND:1–4	4.33	0.19 ± 0.01	0.24	2.9 ± 0.2	95.43	0.01962 ± 0.0007	0.0341	0.6658
BND:1–5	3.05	0.21 ± 0.02	0.18	3.9 ± 0.3	96.77	0.01780 ± 0.0007	0.0307	0.9646
Excitation: 401 nm Emission: 650 nm: MEH-PPV emission.								
BNC:1–1	1.74	0.51 ± 0.02	24.16	0.121 ± 0.002	74.10	0.0336 ± 0.008	0.0630	0.1404
BNC:2–1	1.70	0.71 ± 0.02	22.27	0.180 ± 0.003	76.02	0.049 ± 0.001	0.0895	0.1973
BNC:3–1	2.66	0.52 ± 0.01	28.08	0.135 ± 0.002	69.26	0.037 ± 0.001	0.0775	0.1703
BNC:4–1	2.37	0.58 ± 0.01	26.22	0.158 ± 0.002	71.41	0.050 ± 0.001	0.0909	0.1802
BNC:5–1	2.54	0.57 ± 0.01	36.87	0.144 ± 0.002	60.58	0.050 ± 0.001	0.0983	0.1778
BND:1–1	3.60	0.100 ± 0.002	0.25	0.52 ± 0.01	96.15	0.0013 ± 0.0007	0.0062	0.1673
BND:1–2	5.17	0.081 ± 0.001	0.33	0.401 ± 0.009	94.49	0.0016 ± 0.0008	0.0070	0.1248
BND:1–3	41.80	0.125 ± 0.002	3.42	0.49 ± 0.01	54.78	0.052 ± 0.001	0.0979	0.1676
BND:1–4	46.02	0.130 ± 0.002	4.01	0.48 ± 0.01	49.97	0.058 ± 0.002	0.1080	0.1729
BND:1–5	42.84	0.136 ± 0.002	2.87	0.52 ± 0.01	54.29	0.058 ± 0.001	0.1048	0.1681

a *β*_n_ (%): fractional amplitudes; *τ*_n_ (ns): lifetime; *τ_A_* (ns): amplitude average lifetime; *τ_i_* (ns): intensity average lifetime.

The average fluorescence lifetimes *τ*_*A*_ and *τ*_*i*_ measured at 485 nm for Group C show a significant decrease with increasing PFD’s concentration, strongly suggesting the occurrence of FRET from PFD to MEH-PPV. This observation corroborates the results obtained from the MEH-PPV photoluminescence quantum efficiency measurements and the steady-state fluorescence spectra. Furthermore, the lifetimes *τ*_3_ and *τ*_2_ also decreased systematically with increasing PFD concentration, and the population of the faster lifetime, *β*_3_, increases to nearly 100%. These results indicate that the FRET efficiency increases with the concentration of PFD in the hybrid nanoparticle blends. The progressive shortening of the donor lifetime together with the increase in the contribution of the fastest decay component provides strong time-resolved evidence of efficient nonradiative energy transfer within the hybrid nanoparticle structure.

The fluorescence lifetimes for Group D show a slight increase for longer lifetimes (*τ*_2_) and average lifetimes for the emission at 485 nm, confirming the occurrence of FRET from PFD to MEH-PPV. Moreover, for Group D emission at 650 nm, the shorter lifetime component decreased, whereas the longer component and the average lifetime showed a slight increase. This behavior indicates the presence of additional processes, such as self-absorption and energy migration among MEH-PPV chains, as previously discussed.

From the results of steady-state fluorescence emission, time-resolved fluorescence, and fluorescence quantum yield, we calculate the Förster distance and FRET efficiency and estimate the distance between the donor and acceptor inside the BNC blend samples.

The Förster radius (*R*_0_) was estimated based on the classical expression of FRET theory, which relates the intermolecular distance to the donor quantum yield (*φ*_*d*_), the refractive index (*n*) of the medium in which the polymeric nanoparticles are dispersed (in this case, water), the orientation factor of the transition dipoles (*κ* ^2^ = 2/3), and the spectral overlap integral between the donor emission and the acceptor absorption (Figure S3 supplementary material). Using the donor PLQE of approximately 0.076 (7.6%)^9^ together with the calculated Förster overlap integral (*J*), it was possible to estimate the Förster radius of the system, using the equation below:

$$R_{0}=0.2108{[n^{-4}\varphi_{d}\kappa^{2}J]}^{1/6}$$

The obtained value was 62.5 Å (6.25 nm), indicating a strong spectral overlap and favorable conditions for efficient nonradiative energy transfer between donor and acceptor species. This result suggests that the dipole–dipole coupling between the chromophores involved in the donor–acceptor interaction is significant, enabling efficient energy transfer over nanometer-scale distances.

The efficiency of energy transfer (*E_FRET_*) corresponds to the fraction of absorbed photons by the donor (PFD) that are transferred to the acceptor (MEH-PPV), which can be obtained by the amplitude-weighted lifetime of the donor in the absence (*τ*_*D*_) and in the presence (*τ*_*DA*_) of the acceptor,^34^ by the following relation:

$$E_{FRET}=1-\frac{\tau_{DA}}{\tau_{D}}$$

The donor–acceptor separation distance (*r*) can be estimated using the classic FRET relation based on the Förster radius (*r*_0_) and the calculated FRET efficiency:

$${\left(\frac{r}{R_{0}}\right)}^{6}=\frac{1}{E_{FRET}}-1$$

Unfortunately, this relation tends to overestimate the donor–acceptor distance because we calculated the FRET efficiency using *τ_A_*, which averages the interacting fraction of donors and the noninteracting fraction of the donor. Table 5 presents all the results obtained using the amplitude-weighted lifetime. Therefore, the distances estimated using *τ_A_* should be interpreted as upper-limit values for the donor–acceptor separation within the hybrid nanoparticles.

**Table 5:** FRET Efficiency Results Using the *τ_A_* (Amplitude-Average Lifetime) of the Donor, the Estimated Förster Radius *r*_0_, and the Estimated Distance between the Donor and Acceptor

Samples	*τ*_*A*_ (ns): amplitude average lifetime	*E*_*FRET*_ (%)	(*r*/*R*_0_)^6^	*r* (nm)
BNC:1–1	0.0336	66.57%	0.502	5.57
BNC:2–1	0.0154	84.68%	0.181	4.70
BNC:3–1	0.0105	89.55%	0.117	4.37
BNC:4–1	0.0108	89.25%	0.120	4.39
BNC:5–1	0.0071	92.94%	0.076	4.07
*τ*_*D*_ = 0.10051 ns				***R***_**0**_ **= 6.25 nm**

Table 5 shows the FRET efficiency (*E_FRET_*) increasing and distance (*r*) decreasing for samples with higher concentrations of PFD; however, we believe these results probably represent higher fractions of the donor interacting with the acceptor. Thus, we also calculated the FRET efficiency by separating the interacting fraction of donors and the noninteraction fraction of the donor. We determined the lifetime of the interacting fraction of the donor by analyzing the faster and changing lifetime from the donor–acceptor samples (*τ*_3_) and comparing it with a longer lifetime (*τ*_1_) component that remains similar to the lifetime of the donor alone (since *τ*_2_ is related to excimers and aggregates, it was not considered for this analysis).^21,35^ This approach enables a more direct estimation of the effective donor–acceptor interaction distance:

$$E_{interacting}=1-\frac{\tau_{interacting}}{\tau_{noninteracting}}and{\left(\frac{r}{R_{0}}\right)}^{6}=\frac{1}{E_{interacting}}-1$$

Table 6 shows the results of FRET efficiency of the interacting fraction of the donor and its average distance from the acceptor. The distance between the donor and acceptor was evaluated using the estimated Förster radius (*r*_0_). This approach allows the contribution of noninteracting donor populations to be minimized, providing a more realistic estimation of the effective donor–acceptor separation distance within the hybrid nanoparticles.

**Table 6:** FRET Efficiency for the BNC

Samples	*τ*_1_ (ns) Noninteracting	*τ*_3_ (ns) Interacting	*E*_*interacting*_ (%)	(*r*/*R*_0_)^6^	*r* (nm) Interacting
BNC:1–1	0.25	0.0188	92.48%	0.081	4.11
BNC:2–1	0.23	0.0101	95.61%	0.046	3.74
BNC:3–1	0.15	0.0081	94.60%	0.057	3.88
BNC:4–1	0.20	0.0091	95.45%	0.048	3.76
BNC:5–1	0.11	0.0062	94.21%	0.062	3.93

The donor–acceptor distances obtained using this approach are on the order of ∼3.7–4.1 nm, which is consistent with efficient intraparticle energy transfer between closely packed polymer chains within collapsed hybrid nanoparticle structures. This analysis for BNC samples shows similar distances (*r_interacting_*), with an average of 3.9 ± 0.1 nm and FRET interacting efficiencies (*E_interacting_*), with an average of 94 ± 1 %. Thus, the examination of the results of Tables 5 and 6 indicates that the fraction of donor–acceptor interaction increases with the concentration of PFD while the average distance remains constant.

It is clear that FRET is also present in the BND samples, although the faster lifetime component associated with the 485 emission (PFD fluorescence) rises with the MEH-PPV concentration, indicating that FRET efficiency is decreasing by the increasing amount of MEH-PPV. Moreover, BND samples present lower PLQE and increasing amplitude-weighted lifetime for emission at 650 nm, indicating an intensification in nonradiative processes within the hybrid nanoparticles of Group D. Thus, the ratio between the donor and acceptor is crucial to produce high FRET efficiency that can be translated into higher MEH-PPV quantum yields. Additionally, it is important to emphasize that the lifetime increase for the emission at 650 nm can result from radiative energy transfer, since the photon emitted by the PFD can be absorbed by MEH-PPV that can emit another photon with a slightly higher lifetime. Therefore, efficient FRET in hybrid nanoparticles is maximized when an appropriate donor–acceptor ratio is achieved, ensuring sufficient spectral overlap while maintaining short intermolecular separation distances between interacting polymer chains. Table 7 presents the FRET efficiency using the *τ_A_* (amplitude average lifetime) of the donor and the estimated distance between the donor and acceptor by the classical FRET and using the *τ*_3_ and *τ*_1_ as the lifetime from the interacting fraction of the donor and the noninteracting fraction of the donor obtaining *E*_*interacting*_ and *r*_*interacting*_ for Group D samples. Likewise, Table 7 shows the classical FRET efficiency (*E_FRET_*) decreasing and distance (*r*) increasing for samples with higher concentrations of MEH-PPV, possibly due to lower fractions of the acceptor interacting with the donor. Again, the interacting analysis for BND samples shows similar distances (*r_interacting_*), with an average of 4.31 ± 0.06 nm, and FRET interacting efficiencies (*E_interacting_*), with an average of 90.3 ± 0.7 %, corroborating the lower fraction of donor–acceptor interaction with the increased MEH-PPV concentration and constant average distances.

**Table 7:** FRET Efficiency for the BND

Samples	*E*_*FRET*_ (%)	*r* (nm)	*E*_*interactinginteracting*_ (%)	*r* (nm) Interacting
BND:1–1	80.20%	4.95	90.13%	4.32
BND:1–2	74.23%	5.24	90.04%	4.33
BND:1–3	73.34%	5.28	90.17%	4.32
BND:1–4	66.07%	5.59	89.67%	4.36
BND:1–5	69.46%	5.45	91.52%	4.20

Table 8 summarizes the average hydrodynamic diameters obtained from DLS measurements for Groups C and D. It can be observed that Group D exhibits, on average, smaller hydrodynamic diameters compared to Group C. Thus, increasing the concentration of MEH-PPV tends to maintain the compact hydrodynamic diameters of the nanoparticles, while increasing PFD concentration seems to create less compacted nanostructures (increasing the hydrodynamic diameters and their deviation) and more variability in nanoparticle size distribution. The error bars associated with all measurements represent the mean deviation calculated from multiple independently prepared samples, ensuring greater reliability and reproducibility of the reported data. The slightly larger hydrodynamic diameters observed for Group C are consistent with the formation of hybrid nanoparticles containing both polymer species within the same collapsed structure, which favors shorter donor–acceptor separation distances and consequently enhances FRET efficiency. In contrast, the smaller diameters observed for Group D suggest a different packing arrangement of the polymer chains during nanoparticle formation, which may partially limit donor–acceptor coupling efficiency compared to Group C samples. Consistent with the PLQE and time-resolved spectroscopy results, the hydrodynamic diameter measurements further demonstrate that the order in which the polymers are mixed prior to the formation of the hybrid nanoparticles plays a crucial role in defining their organization and intermolecular interactions. Furthermore, the nearly constant hydrodynamic diameters observed within each group indicate that their characteristic compact structure is preserved, despite the systematic difference in diameter between Groups C and D. These results suggest that increasing the polymer concentration within a given group does not significantly modify the donor–acceptor separation distances. Instead, it changes the fraction of donor–acceptor sets that effectively interact.

**Table 8:** Average Hydrodynamic Diameter Measurements from Groups C and D

Samples	Diameter (nm)	Samples	Diameter (nm)
BNC:1–1	170 ± 26	BND:1–1	138 ± 3
BNC:2–1	205 ± 115	BND:1–2	136 ± 5
BNC:3–1	169 ± 65	BND:1–3	132 ± 14
BNC:4–1	172 ± 38	BND:1–4	138 ± 7
BNC:5–1	192 ± 62	BND:1–5	129 ± 9

Field-emission gun scanning electron microscopy images were obtained for the NCA:1–1 sample and the BNC:1–1 sample of Groups A and C, respectively, as shown in Figure 7. The images confirm the formation of nanoparticles with an approximately spherical morphology. In these images, nanoparticles with characteristic sizes on the order of 40 and 85 nm can be observed for the NCA:1–1 and BNC:1–1 samples, respectively. It should be emphasized that the SEM images are presented only to confirm the formation, size, and morphology of the nanoparticles. SEM does not provide information on the chemical composition of individual nanoparticles and therefore cannot distinguish whether a given nanoparticle consists of PFD, MEH-PPV, or both polymers. Instead, the hybrid nature of the blend nanoparticles is inferred from their photophysical behavior, including the optical spectra, TCSPC, PLQE, and FRET analysis. The average particle sizes observed in the SEM images are consistent with those obtained from DLS measurements, considering the differences between hydrodynamic diameters and dry-state particle dimensions. However, due to the similar electron-contrast response of the two conjugated polymers, it is not possible to distinguish the percentage of each polymer inside each particle from SEM images alone. Nevertheless, the observation of well-defined nanoparticles with sizes consistent with DLS measurements supports the formation of stable hybrid nanoparticle structures containing both polymers within the same nanoscopic domain. These observations are consistent with the efficient donor–acceptor coupling inferred from spectroscopic measurements, indicating that energy transfer occurs predominantly within individual hybrid nanoparticles rather than between separate nanoparticle populations.

**Figure 7:**
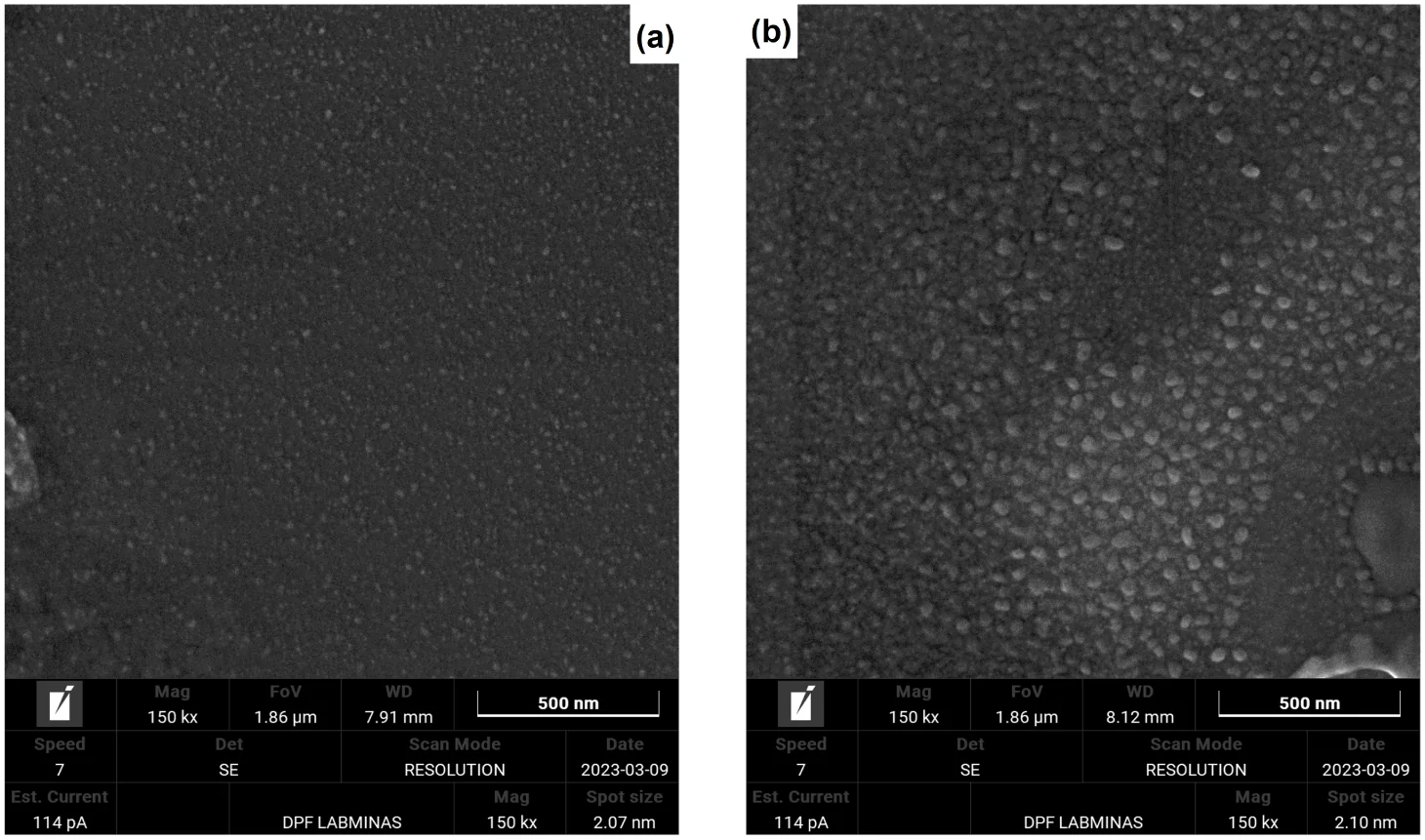
Field-Emission Gun Scanning Electron Microscope (FEG-SEM) image of the sample NCA:1–1 (a) and BNC:1–1 (b) dried and covered with a layer of copper (by sputtering).

## 4 Conclusion

We successfully produced nanocomposites using CPN/MEH-PPV and CPN/PFD and fabricated hybrid blended CPNs based on PFD and MEH-PPV via the reprecipitation method. All these CPNs are predominantly spherical nanoparticles with very similar hydrodynamic diameters, mostly independent of concentration. The nanocomposite groups (A and B) presented optical properties consistent with the independent contributions of CPN/MEH-PPV and CPN/PFD, indicating limited donor–acceptor interaction between separately prepared nanoparticles. Group D hybrid CPN/PFD:MEH-PPV samples exhibited an intensification in nonradiative processes due to the increased MEH-PPV concentration and a less efficient FRET. CPN/PFD:MEH-PPV from Group C presented progressively enhanced FRET between PFD and MEH-PPV, corroborated by a pronounced decrease in PFD’s fluorescence emission, a progressive shortening of the PFD (donor) fluorescence lifetime accompanied by an increased contribution of the faster decay component, a significant increase in PLQE and local donor–acceptor distances approaching 4 nm. In Group C, increasing the PFD concentration boosts FRET efficiency above 90%, thereby enhancing the hybrid nanoparticles' PLQE up to two-fold compared to pure MEH-PPV nanoparticles. These findings demonstrate that the fabrication strategy and donor–acceptor ratio play a decisive role in controlling energy-transfer efficiency overall by changing the fraction of the interacting donor–acceptor in hybrid conjugated polymer nanoparticles, highlighting their potential for applications in luminescent nanostructures, sensing, and photonic devices.

## Supplementary Material





## Data Availability

The data supporting this study’s findings are available from the corresponding author upon reasonable request.

## Notes

Calculations for the FRET efficiencies for BNC and BND samples (XLSX)
Fluorescence decay of the hybrid sample BNC 1:1 with the instrument response function (IRF) and the three-exponential fit of the data, FRET spectral overlap region between the normalized absorption of the acceptor (CPN/MEH-PPV) and normalized emission of the donor (CPN/PFD), absorption and fluorescence emission of the polymers’ solution in THF (HBC and HBD groups), and time-resolved fluorescence parameters for pure PFD and MEH-PPV nanoparticles (PDF)
