# Supplementary material for: Composite and Blends of PFD and MEH-PPV Conjugated Polymer Nanoparticles: Combined Optical Properties and Quantum Yield Increase by FRET
Source: ACS Omega. 2026 Sep 12;11(38):57998–8010. doi: 10.1021/acsomega.6c07561 (PMC13629387; doi:10.1021/acsomega.6c07561)
Supplement: Supplementary file 1 [file ao6c07561_si_002.pdf]

Supporting Information

Composite and Blends of PFD and MEH-PPV Conjugated Polymer Nanoparticles: Combined Optical Properties and Quantum Yield Increase by FRET.

Caio Henrique Viana da Silva[a,*], Milton Lopes de Lana Junior[a,c], Thiago Cazati[b], Mariana P. Brandao[a], Andreza Germana da Silva Subtil[a].

[a]*Departamento de Física, Centro de Ciências Exatas e Tecnológicas, Universidade Federal de Viçosa, Av. P.H. Rolfs, s/n Campus Universitário - 36570-000 - Viçosa – MG – Brazil.*

[b]*Departamento de Física, Instituto de Ciências Exatas e Biológicas, Universidade Federal de Ouro Preto. Campus Universitário Morro do Cruzeiro, CEP 35400-000, Ouro Preto-MG, Brazil.*

[c]*Departamento de Física, Instituto de Ciências Exatas, Av. Antônio Carlos, 6627, CEP 31270-901, Belo Horizonte, MG, Brazil.*

*E-mail: caio.viana@ufv.br.

Fluorescence decay profiles were best described by a triexponential decay model, using deconvolution with the instrument response function (IRF), yielding three lifetime components (τ_1, τ_2, and τ_3) and their corresponding normalized pre-exponential factors (β_1, β_2, and β_3, where $\beta_1 + \beta_2 + \beta_3 = 1$). Figure S1 presents one fluorescence decay of the sample BNC1:1, the IRF and the three exponential fit obtained using FluoFit software.

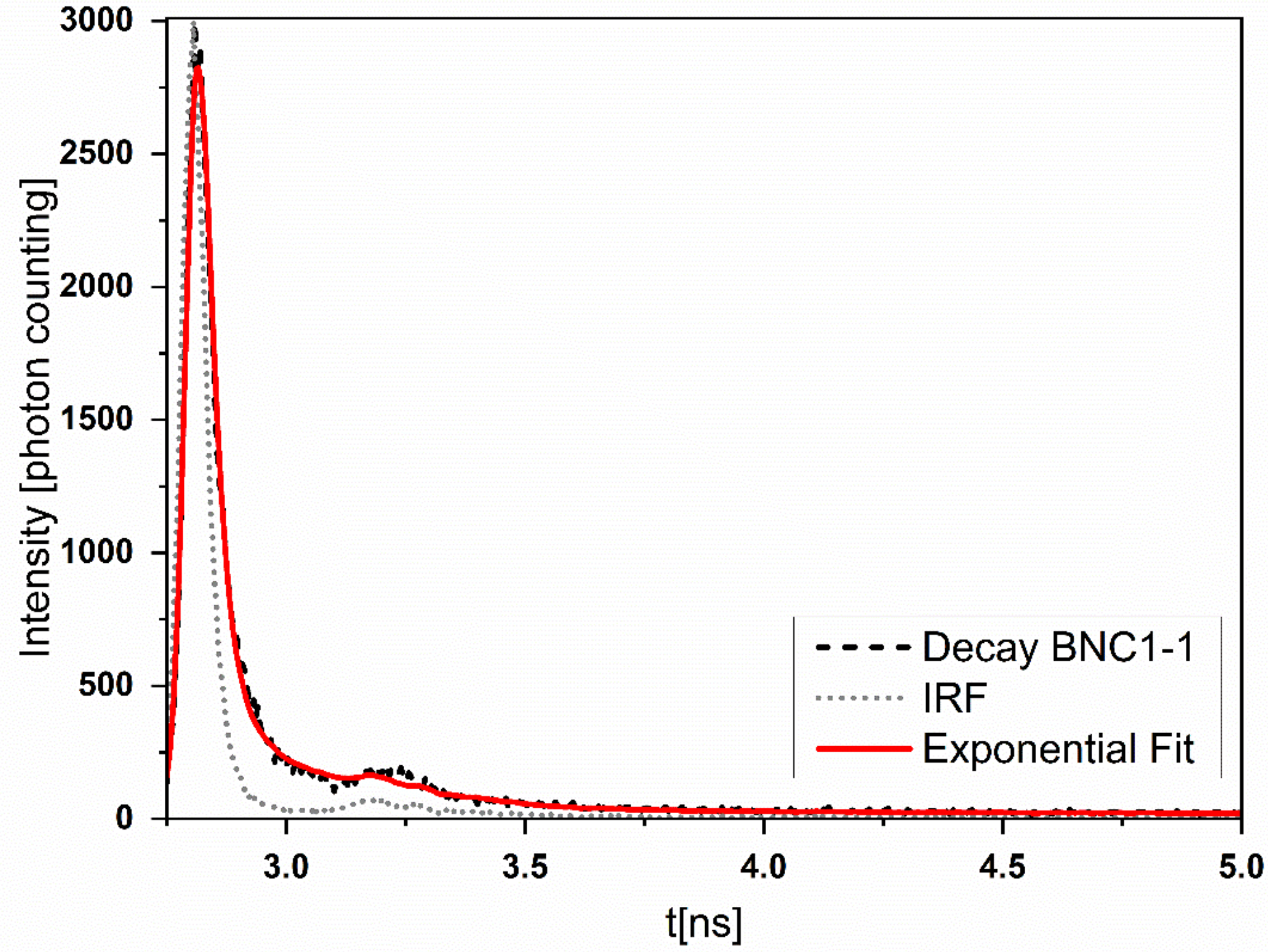

Figure S1: Fluorescence decay of one blend sample BNC1:1, the instrument response function (IRF) and the three-exponential fit of the data.

For the calculus of the Forster distance, we integrate the overlap spectrum of PFD emission (donor) and MEH-PPV absorption (acceptor). Figure S2 shows the overlap region between these two spectra, and we can see that its present a near 100% intersection. This huge overlap area contributes to the high efficiency FRET between PFD and MEH-PPV.

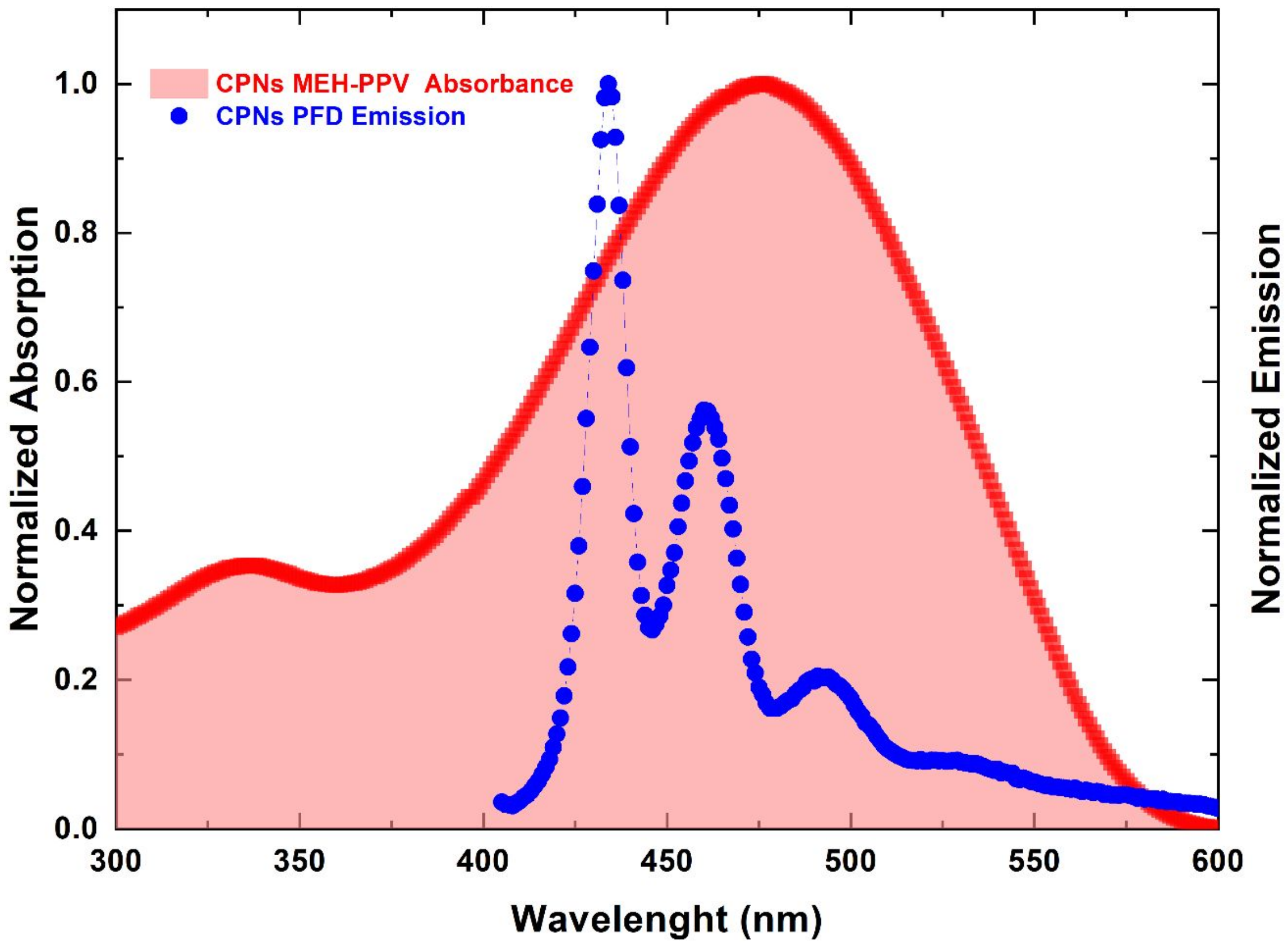

Figure S2: FRET spectral overlap region between the normalized absorption of the acceptor in red (CPN/MEH-PPV) and normalized emission of the donor (CPN/PFD) in blue.

We measured the absorption and fluorescence emission for all hybrid samples (BNC and BND) in the solvent THF (tetrahydrofuran) before the reprecipitation method to form nanoparticles. Figure S3 shows the emission results for all hybrid samples that would form Group C (a and c) and Group D (b and d) in extended form (in THF) before the collapsed state presented in the nanoparticle. It is clear to see the fluorescence of the PFD in the region between 400 and 500 nm, which has higher intensity than MEH-PPV emission (520 nm to 700nm) in this extended form.

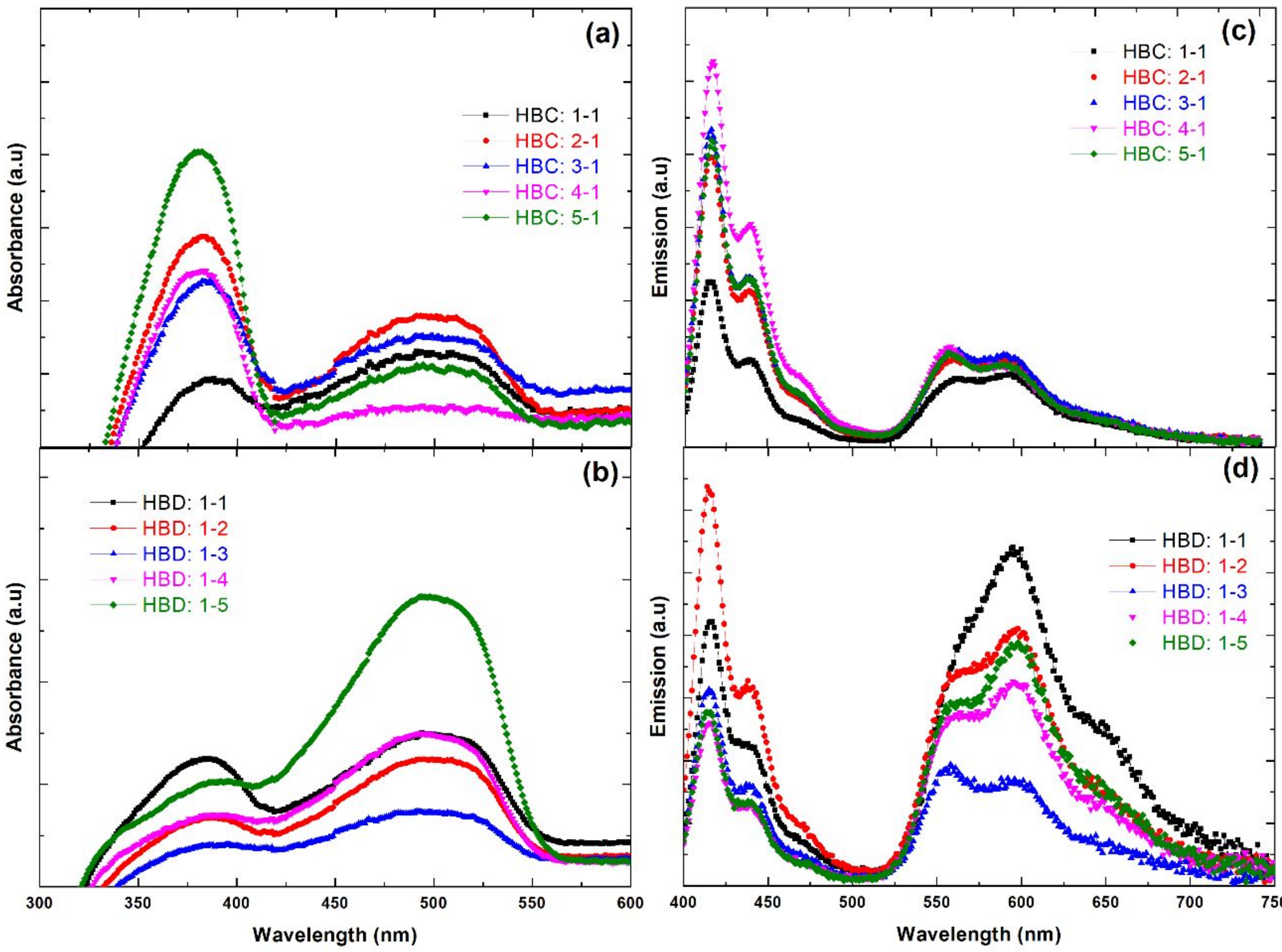

Figure S3: Absorption (a) and Fluorescence (c) emission of the polymers' solution in THF (HBC Group) *before* the creation of CPNs of BNC Group. Absorption (b) and Fluorescence (d) emission of the polymers' solution (HBD Group) in THF *before* the creation of CPNs of BND Group. Excitation at 378nm.

Table S1 provides the values for the lifetimes τ_1, τ_2, and τ_3, the normalized pre-exponential factors β_1, β_2 and β_3, the intensity-weighted fluorescence lifetime τ_i and the amplitude-weighted fluorescence lifetime τ_A for pure CPN/PFD and CPN/MEH-PPV. The reduced chi-squared (χ^2) presented averages values of 0.99±0.01 for CPN/PFD, of 0.97±0.02 for CPN/MEH-PPV.

Table S1: Time-resolved fluorescence parameters for pure PFD and MEH-PPV nanoparticles.

**Samples**	**β_1 (%)**	**τ_1 (ns)**	**β_2 (%)**	**τ_2 (ns)**	**β_3 (%)**	**τ_3 (ns)**	**τ_A (ns)**	**τ_i (ns)**
Excitation: 401 nm Emission: 485 nm: PFD emission.								
**CPN/PFD**	28.36	0.160±0.003	3.60	0.57±0.01	68.04	0.051±0.002	0.1005	0.2053
Excitation: 401 nm Emission: 650 nm: MEH-PPV emission.								
**CPN/MEH-PPV**	30.61	0.120±0.002	2.71	0.45±0.01	66.68	0.032±0.009	0.0707	0.1517

β_n(%): fractional amplitudes; τ_n(ns): lifetime; τ_A (ns): amplitude average lifetime; τ_i (ns): intensity average lifetime.
